# Positive- vs. negative-pressure extubation technique: a scoping review

**DOI:** 10.3389/fmed.2023.1169879

**Published:** 2023-05-12

**Authors:** Jing Liu, Fang Li, Xiangyang Qi, Xin Zhuang, Zhaomei Cui

**Affiliations:** Intensive Care Unit (ICU), Department of Cardiac Surgery, Shandong Provincial Hospital Affiliated to Shandong First Medical University, Jinan, Shandong, China

**Keywords:** airway extubation, extubation complications, extubation methods, positive-pressure extubation, positive-pressure respiration, ventilator weaning

## Abstract

**Objectives:**

This review aimed to summarize the recent literature on positive-pressure extubation.

**Design:**

A scoping review was conducted under the framework of the Joanna Briggs Institute.

**Data sources:**

Web of Science, PubMed, Ovid, Cumulative Index to Nursing & Allied Health, EBSCO, Cochrane Library, Wan Fang Data, China National Knowledge Infrastructure, and China Biology Medicine databases were searched for studies on adults and children.

**Study selection:**

All articles describing the use of positive-pressure extubation were considered eligible for inclusion. The exclusion criteria were articles not available in English or Chinese, and those without full text available.

**Data extraction and synthesis:**

The database searches identified 8,381 articles, 15 of which could be included in this review, with an aggregated patient number of 1,544. Vital signs, including mean arterial pressure, heart rate, R-R interval, and SpO_2_ before and after extubation; blood gas analysis indexes, including pH, oxygen saturation, PaO_2_, and PaCO_2_ before and after extubation; and incidence of respiratory complications, including bronchospasm, laryngeal edema, aspiration atelectasis, hypoxemia, and hypercapnia, were reported in the included studies.

**Results:**

The majority of these studies reported that the positive-pressure extubation technique can maintain stable vital signs and blood gas analysis indices as well as prevent complications during the peri-extubation period.

**Conclusions:**

The positive-pressure extubation technique has a safety performance similar to that of the traditional negative-pressure extubation technique and may lead to better clinical outcomes, including stable vital signs, arterial blood gas analysis, and a lower incidence of respiratory complications.

## Introduction

Extubation is defined as removing the endotracheal tube (ETT), which is the last step in releasing a patient from mechanical ventilation. Extubation can have physiological effects, which may be broadly categorized into cardiovascular and respiratory complications, including hypertension, tachycardia, coughing or bucking, and increases in intracranial and intraocular pressure. It has been reported that the most common complications associated with extubation are laryngospasm (25%), desaturation (22%), and coughing (18%) (1), whereas in ICU patients, the most common complications are hypertension (28.4%), desaturation (24. 1%), and tachycardia (23.7%) (2). These effects may be more significant in young children because of their weak functional residual capacity and high oxygen consumption (3). For example, atelectasis is a common complication after the removal of an ETT in neonates, which increases the extubation failure rate (4).

An operational definition for extubation procedures is lacking, according to previous clinical guidelines (5, 6); however, two techniques are described in the literature. One of these is the conventional extubation technique, which involves placing a suction catheter into the ETT and trachea, emptying the cuff, and removing the ETT with continuous suctioning during the entire procedure [so-called negative-pressure extubation technique (NPET)]. The other is the positive-pressure extubation (PPET) technique, which involves applying positive-pressure through the trachea during cuff emptying and extubation. Theoretically, the airflow passing between the ETT and the larynx during PPET pushes the accumulated subglottic secretions upward so that they can be discharged through the oral cavity.

PPET was proposed based on the concept of improving lung opening and reducing aspiration. Prior studies reported better clinical outcomes with PPET, including but not limited to less desaturation, atelectasis, and coughing (4, 7). However, another investigation showed that more than 80% of practitioners simply applied suction trachea techniques, and that < 5% of practitioners adjusted positive end-expiratory pressure settings during extubation in the UK (8). Similarly, 93.5% of participants in Argentina performed endotracheal suctioning and only 12.5% used positive-pressure during extubation (9). Therefore, there is a gap between experiments and clinical practice.

The objective of this scoping review was to identify the body of existing published research that explicitly mentions PPET vs. NPET.

## Review of the literature

### Methods

This study followed the methodology for scoping reviews described by Arksey and O'Malley (10) and the Preferred Reporting Items for Systematic reviews and Meta-Analyses Extension for Scoping Reviews guidelines. We searched for relevant publications in the PubMed, Web of Science, Ovid, Cumulative Index to Nursing & Allied Health, EBSCO, Cochrane Library, China Biology Medicine, China National Knowledge Infrastructure, and WanFang Data databases. Literature was searched from the respective database inception dates until July 31, 2022. The key search terms were “airway tube,” “tracheal tube,” “endotracheal tube,” “tracheal catheter,” “orotracheal tube,” “tracheostomy,” “decannulation,” “airway extubation,” “extubation technique,” “extubation,” “positive-pressure extubation,” and “negative-pressure extubation.” urthermore, a manual search of the references of all included articles and previous review articles was performed to identify additional studies.

All published randomized controlled trials, cluster randomized trials, crossover trials, non-randomized trials, quasi-randomized trials, and observational studies with controls were included. Studies in English and Chinese were accepted. Conference abstracts and review articles were excluded.

### Study selection

Studies were selected independently by two researchers (Liu and Li). The two researchers compared their lists, and any differences in opinion were resolved by discussion to consensus, or through arbitration by a third researcher (Cui), if necessary.

Data extraction and synthesis were conducted by one researcher (Qi), using standard data-extraction forms. Data extracted included the first author, publication year, country, objects, design, sample size, interventions, reported outcomes, results, and conclusions. Another researcher (Liu) verified the extracted data. We organized the extracted data described above for a qualitative synthesis.

### Patient and public involvement

Patients or the public were not involved in the design, or conduct, or reporting, or dissemination plans of our research.

## Results

**Figure 1:**
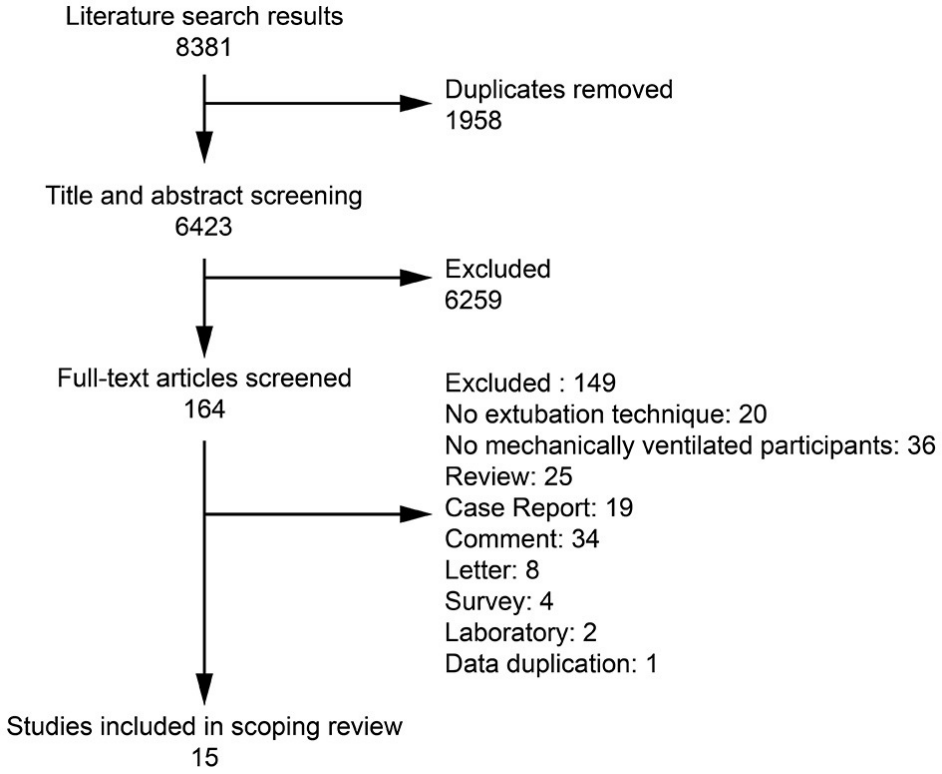
Flow chart of article selection.

In total, 8,381 records were initially identified, of which 1,873 studies were searched in Chinese databases, and 6,508 studies were searched in English databases. After removing duplicates, 6,423 records remained. After reading the titles and abstracts, 164 records that did not meet the inclusion criteria were excluded, and the full texts of eligible articles were further evaluated. Ultimately, 15 publications were included (Figure 1). These articles were all controlled trials, published between April 2008 and March 2022 and are summarized in Table 1.

**Table 1:** Summary of the literature on positive- vs. negative-pressure extubation techniques.

**References, country**	**Subjects**	**Study type**	***N***	**PPET method**	**Reported outcomes**	**Results**
Cai et al. (11), China	Cardiac surgery	Non-RCT	34	1	HR, MAP, saturation of SpO_2_	MAP, HR, and SpO_2_ were more stable in PPET
Cai et al. (12), China	Cardiac surgery	Non-RCT	50	1	MAP, HR, RR, pH, SaO_2_, PaO_2_, PaCO_2_	RR, pH, SaO_2_, PaO_2_, PaCO_2_ had smaller ranges and shorter duration of variation in PPET
Yousefshahi et al. (21), Iran	Cardiac surgery	RCT	252	2	PaO_2_, SpO_2_, pH, PaCO_2_, BE, length of ICU stay, oxygenation index	PPET improved respiratory parameters and attenuated oxygenation complications
Luo et al. (13), China	Infants after cardiac surgery	Non-RCT	50	1	MAP, HR, SpO_2_, pH, PaO_2_, PaCO_2_, incidence of respiratory complications.	Hemodynamics and blood gas analysis indexes were more stable in PPET with lower incidence of complications
Zhang and Jiang (14), China	Infants after cardiac surgery	RCT	80	1	MAP, HR, SpO_2_	MAP, HR, and SpO_2_ were more stable in PPET
Jing et al. (15), China	Emergency ICU	RCT	436	1	SpO_2_, pH, SaO_2_, PaO_2_, PaCO_2_, incidence of respiratory complication, length of ICU stay	PPET can prevent hypoxemia during extubation, decrease the incidence of aspiration and length of ICU stay
Huang et al. (16), China	Infants after cardiac surgery	RCT	80	1	SpO_2_	PPET can prevent SpO_2_ decrease during extubation
Wang and Luo (17), China	Infants after cardiac surgery	RCT	50	1	SpO${}_{2}^{,}$ incidence of respiratory complication	PPET can prevent the decrease of SpO_2_ and respiratory complication
L'Hermite et al. (18), France	Elective orthopedic surgery	RCT	68	1	The time-span between extubation and SpO_2_ to decrease < 92% (T92), and the rate of desaturation < 92%	PPET did not postpone the beginning of desaturation (< 92%), or decrease the demand for supplemental oxygen therapy
Andreu et al. (7), Argentina	Emergency ICU	RCT	236	3	Postextubation overall complications, postextubation pneumonia, extubation failure, reintubation, use of NIV, lenth of ICU stay	PPET does not lead to a higher occurrence rate of complications without requiring more medical devices
Xu et al. (22), China	ICU	RCT	48	4	Change of end expiratory lung impedance (ΔEELI%), Complications within 30 min after extubation	PPET is not time-consuming and simple, it can effectively reduce lung collapse, upper respiratory complications, and vital sign changes caused by extubation
Fei et al. (20), China	ICU	RCT	60	3	HR, RR, DBP, SBP, SpO_2_, incidence of respiratory complication, extubation failure	PPET has less effect on HR, RR, DBP, SBP, and SpO_2_ during extubation, with a lower incidence of complications
Andreu et al. (19), Argentina	Emergency ICU	RCT	725	3	Postextubation major complications, minor complications, postextubation pneumonia, extubation failure, length of ICU stay	PPET reduced the rate of major complications and minor complications, without statistically significant differences
Farhadi et al. (4), Iran	Newborns	RCT	100	1	PEA, duration of ventilator therapy, extubation failure, rate of pneumothorax, apnea, death within 72 h after extubation	PPET decreased the onset of PEA and incidence rate of extubation failure
Liu et al. (23), China	ICU	RCT	105	4	pH, PaO_2_, PaCO_2_, complications during extubation, pneumonia within 48 h after extubation.	PPET can guarantee adequate oxygenation. It can also decrease the

PPET Method: 1. Connect a resuscitation capsule filled with oxygen and equipped with a pressure gauge to the ETT; 2. Machenical ventiliation in PSV mode, with an inspiratory pressure of 20 cm H_2_O and PEEP of 15 cm H_2_O; 3. Machenical ventiliation in PSV mode, with an inspiratory pressure of 15 cm H_2_O and PEEP of 10 cm H_2_O; 4. Machenical ventilation in SBT mode with no specific description.

PPET, positive-pressure extubation technique; NPET, negative-pressure extubation technique; HR, heart rate; MAP, mean arterial pressure; RR, R-R interval; ETT, endotracheal tube; PSV, pressure support ventilation; BE, base excess; NIV, non-invasive ventilation; DBP, diastolic blood pressure; SBP, systolic blood pressure; PEA, postextubation-atelectasis; SBT, spontaneous breath test.

### Positive-pressure extubation technique

In the 15 included studies, PPET was used in 1,032 adults in the ICU, 444 infants, and 68 adults emerging from anesthesia. All the included studies reported the effects of PPET vs. NPET. PPET and NPET were implemented in the same way, using three different methods. One of the three PPET methods was reported in nine studies (4, 11–18); it involved connecting a resuscitation capsule filled with oxygen and equipped with a pressure gauge to the ETT during the inspiration phase. Seven studies published between 2008 and 2016 were conducted on patients after cardiac surgery in China (11–17). One study was conducted on patients emerging from anesthesia in France (18). The remaining study was conducted on newborns undergoing mechanical ventilation in Iran (4). Another method involved removing the ETT at the end of the inspiratory period, with a higher PEEP, and ventilator parameters were set to pressure support ventilation mode, which was reported in four studies and all of them were conducted on ICU patients [two in Argentina (7, 19), one in China (20), and one in Iran (21)]. The last method involved removing the ETT at the end of the inspiratory period under the original ventilation mode, which was reported in two studies (22, 23), all of which were conducted on ICU patients in China.

### Clinical performance indicators

Vital signs, including mean arterial pressure, heart rate, respiratory rate, and SpO_2_ before and after extubation; blood gas analysis indexes including pH, oxygen saturation, PaO_2_, and PaCO_2_ before and after extubation. The incidence of respiratory complications, including bronchospasm, laryngeal edema, aspiration atelectasis, hypoxemia, and hypercapnia were reported in the included studies. The majority of these studies reported that PPET can maintain stable vital signs and blood gas analysis indices, as well as prevent complications during the peri-extubation period. Nevertheless, one study (18) showed that PPET did not appear to delay the onset of hyposaturation or reduce the need for oxygen therapy during the first 10 min after extubation, but its patients underwent short-term ventilation. In addition, the most significant differences between the two groups came from small- sample studies, and the largest multicenter study (19) showed no significant differences between NPET and PPET groups. Four studies reported extubation failure of the two techniques. Two (4, 7) reported a significant decrease in extubation failure, and the other two (19, 20) reported no significant differences.

## Discussion

In theory, PPET has potential advantages over NPET, based on the concept of lung opening and reducing aspiration to maximize alveolar recruitment. A study on newborns has confirmed that compared to NPET, PPET reduces the rate of post-extubation atelectasis (24), and positive-pressure ventilation with oxygen can be provided with a bag-valve device during endotracheal extubation.

However, because secretions accumulate in the subglottic space during invasive mechanical ventilation, they can be aspirated into the airways during cuff deflation and tracheal extubation. This may not be associated with clinical manifestations at the time but may lead to pneumonitis or pneumonia. In laboratory research (25), negative suctioning during cuff deflation and extubation was shown to result in increased leakage into the lower respiratory tract. This could be addressed by mechanical ventilation using the pressure support mode at a level of 15/10 cm H_2_O or 20/5 cm H_2_O, resulting in minimal leakage. In another laboratory study (26), three extubation methods were designed to measure the leakage of water or artificial sputum (with different viscosities): negative-pressure suction, positive-pressure by a resuscitator, and ventilation following a continuous positive airway pressure mode with the pressure level set at 5, 10, and 20 cm H_2_O. The results showed that continuous positive airway pressure extubation resulted in less secretion leakage than the other methods. In terms of the different positive pressure levels, the optimal pressure at 5 cm H2O resulted in a smaller amount of secretion leakage, even when viscosity was higher.

In this first scoping review of positive vs. NPETs, we found that PPET was not inferior to NPET, based on the current literature, which may maintain stable vital signs and blood gas analysis indexes, as well as prevent complications during the peri-extubation period in both adults and infants undergoing mechanical ventilation. However, because the settings, patients, and methods of PPET in different studies are inconsistent, and there is a relative risk of publication bias. Furthermore, the use of PPET by adjusting the PEEP level on the ventilator has been shown to save manpower during the extubation process, without increasing safety risks (7). However, this has not been investigated in infants in the literature, and it is important to determine the appropriate ventilator parameters. Extubation failure is an important indicator related to the clinical outcome of patients. Considering that the extubation failure rate is related to many factors, such as disease status, implementation of spontaneous breathing tests and extubation procedures, further clinical trials are needed to verify the effect of PPET on extubation failure.

Moreover, all the outcomes reported in the literature to date reflect short-term effects, with no longer than 72 h of follow-up. Long-term indicators, such as length of hospital stay and cost, did not differ between PPET and NPET.

This study had some limitations. Owing to our review method, we may have missed publications written in languages other than English or Chinese. Considering the heterogeneity of the present PPET articles on subjects, settings, different ways of extubation, differences in follow-up and reporting of results based on the present literature; it is difficult to draw definitive conclusions about the efficacy of PPET. Thus, this article is written as a scoping review to describe the current evidence to identify directions for future research. Given this, randomized controlled trials and cohort studies that compare different methods and pressure levels of PPET, particularly in infants and pediatric patients are needed to answer all clinical questions and verify its safety.

## Conclusion

The current literature suggests that PPET is as safe as NPET and may lead to better clinical outcomes, including stable vital signs, improved arterial blood gas analysis results, and a reduced incidence of respiratory complications during extubation. Clinical trials and cohort series with uniform operating standards are required to explore the safety and efficacy of PPET further.

## Data availability statement

The original contributions presented in the study are included in the article/supplementary material, further inquiries can be directed to the corresponding author.

## Author contributions

JL and ZC contributed to study design and conceptualization. FL and JL contributed to databases searching. XQ and XZ contributed to data screening and data extraction. JL and XZ contributed to data synthesis, interpretation, and manuscript drafting. ZC and FL contributed to critical revision. All authors contributed to the article and approved the submitted version.
